# Oestrogen in the chick embryo can induce chromosomally male ZZ left gonad epithelial cells to form an ovarian cortex that can support oogenesis

**DOI:** 10.1242/dev.181693

**Published:** 2020-02-25

**Authors:** Silvana Guioli, Debiao Zhao, Sunil Nandi, Michael Clinton, Robin Lovell-Badge

**Affiliations:** 1 The Francis Crick Institute, London, NW1 1AT, UK; 2 The Roslin Institute and R(D)SVS, Gene Function and Development, University of Edinburgh, Edinburgh, EH25 9RG, UK

**Keywords:** Ovary differentiation, Oestrogen, Gonadal chimera, Chicken embryo, Sex determination

## Abstract

In chickens, the embryonic ovary differentiates into two distinct domains before meiosis: a steroidogenic core (the female medulla), overlain by the germ cell niche (the cortex). The differentiation of the medulla is a cell-autonomous process based on chromosomal sex identity (CASI). In order to address the extent to which cortex differentiation depends on intrinsic or extrinsic factors, we generated models of gonadal intersex by mixing ZW (female) and ZZ (male) cells in gonadal chimeras, or by altering oestrogen levels of ZW and ZZ embryos. We found that CASI does not apply to the embryonic cortex. Both ZW and ZZ cells can form the cortex and this can happen independently of the phenotypic sex of the medulla as long as oestrogen is provided. We also show that the cortex-promoting activity of oestrogen signalling is mediated via estrogen receptor alpha within the left gonad epithelium. However, the presence of a medulla with an ‘intersex’ or male phenotype may compromise germ cell progression into meiosis, causing cortical germ cells to remain in an immature state in the embryo.

## Abstract (precis)

**Summary:** Cell-autonomous sex identity does not apply to chicken ovarian cortex development. The gonadal epithelium responds to oestrogen via ERα and drives cortex formation independently of the medulla's phenotypic sex.

## INTRODUCTION

The gonads provide a paradigm in which to investigate organogenesis given that there are two possible outcomes, ovaries and testes, which arise from a common anlagen and cell lineages.

In the chick, gonadal sex-specific morphological differences become grossly apparent from around day (D) 8 (HH34) (Hamburger and Hamilton, 1951) with the localisation of the germ cells to the appropriate domain. In chromosomally male (ZZ) embryos the germ cells become embedded within somatic cells to form the sex cords of the gonadal core or medulla, whereas in chromosomally female (ZW) embryos the germ cells aggregate at the periphery of the gonad and intermingle with somatic cells to form the cortex (Carlon and Stahl, 1985). In the embryonic phase of ovary differentiation, the germ cells organise in nests. It is only after hatching, when the cortex undergoes major restructuring, in a process known as folliculogenesis, that the nests are broken and each germ cell becomes enveloped by layer(s) of granulosa cells. The embryonic cortex is therefore transient, but it is a common feature of most amniotes, including many mammals. It precedes the entry of germ cells into meiosis. Mouse and rat are exceptions to this rule, because their germ cells remain distributed in the whole gonad long after meiotic entry (Byskov, 1986; DeFalco and Capel, 2009).

The formation of the embryonic cortex is side dependent in the chick. This is due to the interaction of the canonical left-right asymmetry pathway with the gonadal development pathway via the transcription factor *Pitx2*, expressed asymmetrically in the left gonadal epithelium (Guioli and Lovell-Badge, 2007; Ishimaru et al., 2008; Rodriguez-Leon et al., 2008). During sex-specific differentiation of the gonads, this asymmetry is ignored or overwritten in males, whereas it is exacerbated in females. Indeed, at sex determination, the medulla of both left and right embryonic ZW gonads differentiate into steroidogenic domains, but only the left gonad develops a cortex and proceeds to differentiate further into a functional ovary; the right ovary remains just as a steroidogenic organ and later atrophies. Moreover, all the germ cells in the right ovary are scattered in the medulla, do not properly enter meiosis and are lost post-hatching, a fate shared with the germ cells remaining in the medulla of the left ovary (Guioli et al., 2014). This asymmetry points to the gonadal epithelium as pivotal in the formation of the ovarian cortex and establishes the importance of the embryonic cortex for survival and maturation of the germ cells.

Recent chimera studies have demonstrated that somatic cells of the chick gonad medulla differentiate into either testicular cells or ovarian steroidogenic cells in a cell-autonomous manner, i.e. dependent on their sex chromosome constitution (Zhao et al., 2010), a phenomenon known as cell-autonomous sex identity (CASI). However, the importance of intrinsic sex chromosome identity in the formation of the cortical germ cell niche has yet to be addressed.

Like lower vertebrates and many mammals, although not the mouse, the embryonic ovaries of birds produce oestrogen from the start of female-specific differentiation. For chicken and some other vertebrate species, including some mammals, it has been shown that ectopic manipulation of oestrogen levels at the time of sex determination can override the effects of chromosomal sex. Notably, blocking the synthesis of oestrogen in ZW embryos before sex differentiation results in female-to-male sex reversal. In these birds, both the left and right medulla start to express male markers, such as *SOX9*, in differentiating cords and the left epithelium fails to make a cortex. Conversely, injecting oestrogen before sex differentiation in ZZ embryos results in male-to-female sex reversal. Although this phenotype is reported to revert to normal in the adult, in the embryo both medullas express *FOXL2* and *CYP19A1* and a cortex develops on the left side (Akazome and Mori, 1999; Bruggeman et al., 2002; Vaillant et al., 2003; Yang et al., 2008).

During chick sex determination, estrogen receptor alpha (ERα; ESR1) is expressed in both the left and right medulla, but asymmetrically in the epithelium of the left gonad (Andrews et al., 1997; Guioli and Lovell-Badge, 2007). This makes it a good candidate for the oestrogen transducer, with the hypothesis that oestrogen affects the differentiation of both medulla and cortex by acting on different cell types and different pathways. Furthermore, it suggests once again the pivotal role of the epithelium in the formation of the cortex.

In order to understand the process of embryonic cortex morphogenesis, we investigated the importance of oestrogen signalling in cortex differentiation in relation to the chromosomal sex of gonadal cells. By following the fate of mixed sex gonadal chimeras and of gonads derived from embryos with manipulated oestrogen levels, we show that cortex formation is not a CASI process and that oestrogen is the only signal necessary for induction. However, the progression of cortical germ cells to meiosis is compromised in gonadal intersex phenotypes. Finally, we show that downregulating epithelial ERα is sufficient to affect cortex differentiation severely, indicating that epithelial ERα is the relevant signal transducer.

## RESULTS

### Modifying oestrogen levels after the point of sex determination affects cortex formation without affecting the sex identity of the medulla

**Fig. 1.**
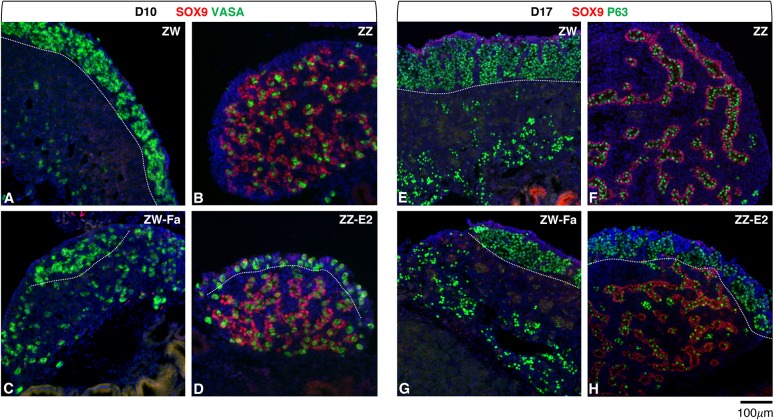
**Perturbing oestrogen levels at embryonic D7-7.5 (HH31) affects cortex formation in ZW and ZZ embryos.** (A-H) Sections from left gonads at D10 (HH36) (A-D) or D17 (HH43) (E-H) double-stained for the Sertoli marker SOX9 (red) and a germ cell marker (VASA or P63; green) in ZW controls (A,E), ZZ controls (B,F), ZW gonads treated with fadrozole (ZW-Fa) (C,G) and ZZ gonads treated with β-oestradiol (ZZ-E2) (D,H). Decreasing oestrogen in ZW embryos after sex determination compromises the differentiation of the ovarian cortex; adding β-oestradiol in ZZ embryos after sex determination induces the formation of a cortex on top of a male medulla. White dotted lines highlight the cortex-medulla border.

In order to understand the role of oestrogen in cortex differentiation and the relationship between sex-specific differentiation of cortex and medulla, we altered oestrogen levels beyond the time when sex reversal can be achieved (Bruggeman et al., 2002). To block/reduce oestrogen levels we injected D7-7.5 (HH31) ZW embryos with the aromatase inhibitor fadrozole and repeated the treatment every 2 days (ZW-Fa embryos) (Fig. 1). Gonads recovered at D10 (HH36) showed a female medulla as expected, with no sign of masculinisation, as no male markers such as SOX9 were identified by immunostaining, similar to the ZW wild type. However, the cortical domain of the left ovaries appeared to be smaller compared with controls and contained fewer germ cells (Fig. 1A-C). ZW left ovaries collected at D17 (HH43) were morphologically much smaller compared with ZW controls (Fig. S1), but still had a cortical domain. However, this was generally limited to the central part of the ovary (Fig. 1E-G).

To upregulate oestrogen in ZZ embryos after sex determination, we injected β-oestradiol *in ovo* at D7-7.5 (HH31) (ZZ-E2 embryos) (Fig. 1). The resulting ZZ left gonads collected at D10 (HH36) comprised a male medulla containing cords made of SOX9-positive somatic cells and germ cells, overlain by a narrow cortex-like domain (Fig. 1A,B,D). Left and right gonads recovered at D17 (HH43) showed a more striking phenotype, with a well-developed male medulla on both sides and a thick cortical domain on the left side, containing germ cell nests (Fig. 1E,F,H). Similar results were obtained when β-oestradiol was injected much later [i.e. at D9 (HH35); Fig. S2].

These results show that the early differentiation of the cortex can be independent of the sex of the medulla and points to oestrogen as the key inducer of this process.

### ZW and ZZ cells can contribute to the cortical domain in mixed-sex gonadal chimeras

**Fig. 2.**
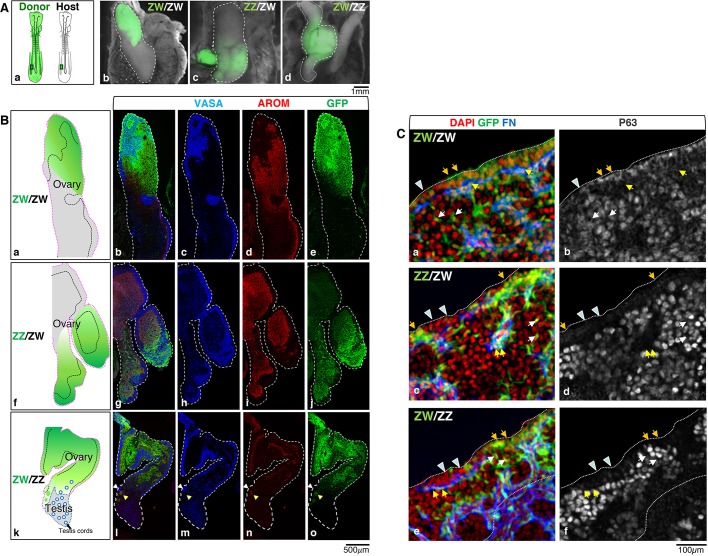
**ZW and ZZ cells can both contribute to the somatic component of the cortex domain.** (Aa) Schematic illustrating the transplant procedure. D18 (HH44) gonadal chimeras were generated by transplanting posterior lateral plate/intermediate mesoderm from the left side of donor GFP embryos (green) into the left side of recipient wild-type embryos at D1.5-2 (HH10). (Ab-Ad) Whole-mount images of the resulting gonads dissected at D18 (HH44) showing the chimeric left gonad enriched in donor GFP (green) cells in ZW donor into ZW host (ZW/ZW) (Ab), ZZ donor into ZW host (ZZ/ZW) (Ac) and ZW donor into ZZ host (ZW/ZZ) (Ad). White dashed and dotted lines highlight the chimeric left gonad (ovarian and testis domain, respectively). (Ba-Bo) Immunofluorescence images of sections from the left chimeric gonads showing the germ cells using the marker VASA (blue), the female-specific steroidogenic cells using the marker P450 aromatase (AROM; red) and the GFP-positive donor cells (green) in ZW/ZW control chimera (Ba-Be), ZZ/ZW chimera (Bf-Bj) and ZW/ZZ chimera (Bk-Bo). The schematics summarise the immunostaining results (see also Fig. S3), showing the ovarian domain (pink dotted line border), the cortical germ cells nests (superficial areas delimited by black dashed lines), the testis domain (blue dotted line) and the GFP-enriched area (green). ZW/ZW (Ba-Be) and ZZ/ZW (Bf-Bj) chimeras are ovaries, and the ZW/ZZ (Bk-Bo) is an ovotestis. White arrowheads indicate a small cortical germ cell nest overlaying a patch of AROM donor cells (yellow arrowheads) in the transition area between ovary and testis. (Ca-Cf) High-resolution images of cortical areas from ZW/ZW, ZZ/ZW and ZW/ZZ chimera sections immunostained for the extracellular matrix protein Fibronectin 1 (FN; blue) and the germ cell marker P63 (grey); nuclei are counterstained with DAPI (red). GFP (green) can be found in the cortex epithelium (orange arrows) and in somatic cells close to the germ cells nests, either positive (yellow arrows) or negative (white arrows) for FN. The ZZ donor cells in the ZZ/ZW chimera (Cc,Cd) and the ZW donor cells in the ZW/ZZ chimera (Ce,Cf) can both contribute the somatic components of the cortical domain. Epithelial donor cells (orange arrows) and host cells (arrowheads) may intermingle. Dotted lines delineate cortex borders.

It has previously been shown that, in the chick, the medullary fate is determined by CASI (Zhao et al., 2010). As the differentiating embryonic ovary is composed of two distinct sex-specific functional domains – a steroidogenic medulla and a cortical domain essential for germ cell maturation – it remains to be assessed how important cell-autonomous sex identity is for the formation and differentiation of a proper ovarian germ cell niche. Oestrogen manipulation experiments have demonstrated that a cortex can form in a ZZ embryo; however, it was not clear whether this is possible under normal physiological conditions. To address this issue, we generated gonadal chimeras comprising male and female cells by transplanting D2 (HH10) lateral plate/intermediate mesoderm from the left side of donor GFP embryos into age-matched and same-side recipient embryos of the same or opposite sex, *in ovo*. The manipulated embryos were re-incubated and the gonads were collected at D18 (HH44) (Fig. 2A) and the cortex cellular composition was examined.

The resulting manipulated embryos contain a host-derived right gonad and a left gonad containing somatic cells of host and donor origin. The germ cells, however, are always host derived on both sides, as they segregate to the germinal crescent of the embryo at stage HH5 and later migrate via the bloodstream to reach the intermediate mesoderm at HH15 (Ginsburg and Eyal-Giladi, 1987; Karagenc et al., 1996; Nakamura et al., 1988, 2007).

The chimeric gonads derived from left-to-left transplants between ZW embryos (ZW/ZW, same-sex chimeras) were ovaries composed of a medulla and a cortex similar to those in a normal left ovary. In the donor-enriched area, the cortical epithelial and sub-epithelial somatic cells were mainly contributed by GFP donor cells (Fig. 2Ba-Be,Ca,Cb, Fig. S3A).

Fig. 2Bf-Bj and Fig. S3B show a typical chimeric left ovary from a ZW embryo containing donor cells derived from ZZ left mesoderm (ZZ/ZW mixed-sex chimera). This ovary displays a cortex along the entire length. As expected from previous studies (Zhao et al., 2010), the area enriched in donor tissue was composed of a medulla containing host (ZW), P450 aromatase (CYP19A1)-positive steroidogenic cells, surrounded by interstitial cells derived from both host (ZW, GFP negative) and donor (ZZ, GFP positive). Surprisingly, in the GFP-enriched area, the medulla was overlain by a cortex formed by GFP somatic donor (ZZ) cells. These areas were continuous with areas poor in donor cells, where medulla and cortex were mainly host derived. At the junction between the two areas, intermingling of host and donor cells of the cortex was evident (Fig. 2Cc,Cd). Transplants of ZW donor tissue into ZZ hosts (ZW-ZZ mixed-sex chimeras) often produced chimeric ovotestis. Fig. 2Bk-Bo,Ce,Cf and Fig. S3C show one such example. The ovarian portion of the chimera comprised a female medulla containing P450 aromatase-positive steroidogenic cells derived from the donor (ZW, GFP-positive cells) as expected, overlain by a cortex made of both host (ZZ) and donor (ZW) somatic cells. This ovarian domain was contiguous with a testicular domain (Fig. 2Bk-Bo, Fig. S3C).

We concluded that both ZZ and ZW cells can normally contribute the somatic component of the embryonic cortex without a sex bias and therefore independently of their chromosomal sex. Therefore, it is clear that, although medullary fate is determined by CASI (Zhao et al., 2010), this concept does not apply to the gonadal cortex.

### Meiotic entry of cortical germ cells is severely compromised in gonads with a masculinised medulla

The mitotic-to-meiotic switch is asynchronous in the chicken ovarian cortex, with many germ cells entering meiosis by D12 (HH38) and the majority initiating the synaptic process by D16 (HH42) (de Melo Bernardo et al., 2015).

**Fig. 3.**
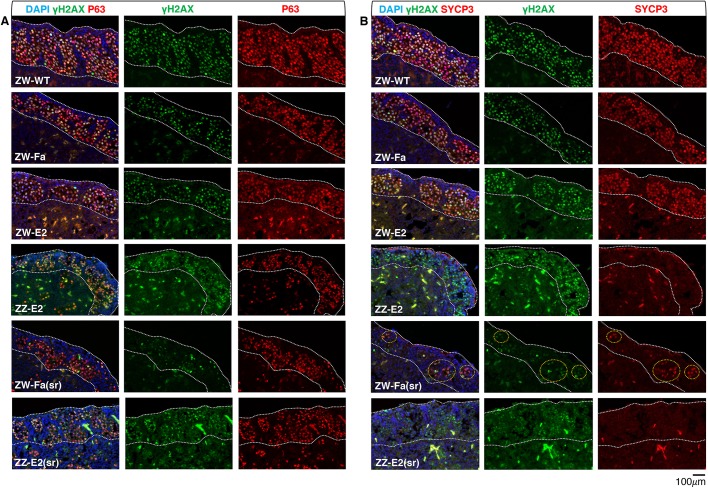
**Induction of meiosis is compromised in the cortical germ cells of D17 embryos subject to oestrogen level alterations.** (A) D17 (HH43) left gonad sections immunostained for γH2AX (green) and P63 (red); nuclei are counterstained with DAPI (blue). (B) Sections from the D17 (HH43) left gonad shown in A immunostained for γH2AX (green) and SYCP3 (red). ZW-WT, ZW wild type; ZW-Fa, ZW treated with fadrozole from D7-7.5 (HH31); ZW-E2, ZW treated with β-oestradiol at D7-7.5; ZZ-E2, ZZ treated with β-oestradiol at D7-7.5; ZW-Fa(sr), ZW, partially sex-reversed gonad, treated with fadrozole at D4; ZZ-E2(sr), ZZ, partially sex-reversed gonad, treated with β-oestradiol at D4. All gonadal models have a cortical domain containing germ cells. In ZW-Fa ovary and ZW-E2 ovary, most cortical germ cells express SYCP3 like the ZW-WT control. In ZW-Fa(sr) ovotestis, very few germ cells express γH2AX and SYCP3 (orange dotted circled areas). In ZZ-E2(sr) ovotestis and ZZ-E2 testis overlain by a cortex, some cortical germ cells express γH2AX but none expresses SYCP3. White dotted line highlights the cortical domain borders. See Fig. S4 for the medullary structure of the ZZ-E2, ZW-Fa(sr) and ZZ-E2(sr) models.

We followed germ cell meiotic progression in D17 (HH43) ovaries from ZW embryos subject to late fadrozole treatment (ZW-Fa) and from ZZ embryos subject to late oestradiol treatment (ZZ-E2), by analysing the expression of the double-strand break (DSB) marker γH2AX and the synapsis marker SYCP3 (Guioli et al., 2012; Smith et al., 2008). At this stage, both markers are normally expressed in most germ cells of the wild-type ovary (Fig. 3). Similarly, many germ cells expressed both markers in the oestradiol-treated ZW left gonads (ZW-E2). In fadrozole-treated ZW left gonads (ZW-Fa) the cortex contained fewer germ cells, but was positive for γH2AX and SYCP3. Meiosis was most affected in oestradiol-treated ZZ left gonads (ZZ-E2), composed of a medulla organised in testicular cords (Fig. S4), overlain by a cortical domain made of ZZ somatic cells and ZZ germ cells: some cortical germ cells expressed γH2AX but none was positive for SYCP3 as assessed by immunostaining (Fig. 3B).

To investigate the importance of germ cell chromosomal sex for the correct initiation of meiosis in the cortex, we generated a series of testis-to-ovary reversed ZZ embryos and a series of ovary-to-testis reversed ZW embryos, by *in ovo* injection of oestradiol or fadrozole, respectively, at D4 (HH23) [ZZ-E2(sr) and ZW-Fa(sr) embryos]. From both experiments, we obtained D17 embryos with incomplete gonadal sex reversal that typically showed an ‘intersex’ phenotype. In the left gonad, the inner part of the medulla contained SOX9-positive cells, organised in cord-like structures, whereas the outer medulla contained more FOXL2-positive cells (Fig. S4). The medulla was generally overlain by a cortex like structure (Fig. 3A). Importantly, in ZZ-E2(sr) embryos the primordial germ cells (PGCs) are male (ZZ), whereas in ZW-Fa(sr) embryos they are female (ZW). Synapsis was compromised in both these models, because in both cases some cortical germ cells expressed γH2AX but none or very few expressed SYCP3 as determined by immunostaining (Fig. 3).

**Fig. 4.**
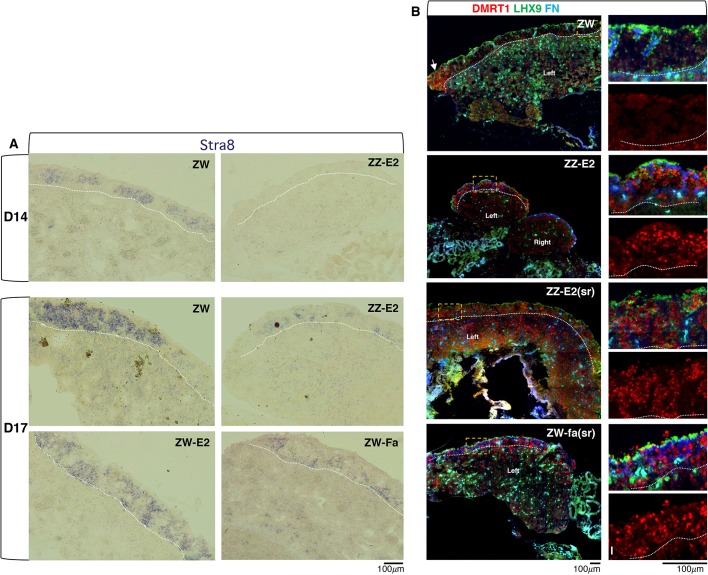
**The** **expression pattern of mitotic-meiotic switch markers is affected in the cortical germ cells of embryos subject to oestrogen levels alteration.** (A) RNA *in situ* analysis of *STRA8* expression on sections from the left gonad of D14 (HH40) and D17 (HH43) embryos. ZW, female wild-type control; ZZ-E2, ZZ treated with β-oestradiol at D7-7.5 (HH31); ZW-E2, ZW treated with β-oestradiol at D7-7.5 (HH31); ZW-Fa, ZW treated with fadrozole at D7-7.5 (HH31). *STRA8* expression is severely compromised in the gonadal cortical germ cells from ZZ embryos exposed to β-oestradiol. (Ba-Bl) Immunofluorescence detection of DMRT1 (red), LHX9 (green) and Fibronectin 1 (FN; blue) on gonadal cryostat sections from the following D17 (HH43) embryos: ZW, female wild-type control (Ba-Bc); ZZ-E2, ZZ treated with β-oestradiol at D7-7.5 (HH31) (Bd-Bf); ZZ-E2(sr), ZZ treated with β-oestradiol at D4 (HH23) (partially sex reversed) (Bg-Bi); ZW-Fa(sr), ZW treated with fadrozole at D4 (HH23) (partially sex reversed) (Bj-Bl). Right-hand panels show high-magnification images of the boxed areas on the left; (Bb,Be,Bh,Bk) LHX9, FN and DMRT1; (Bc,Bf,Bi,Bl) DMRT1 only. All samples were cut sagittally (left gonad shown) apart from ZZ-E2, which was cut transversally (left and right gonads shown). White dotted lines mark the cortex-medulla border. In the left wild-type ovary, LHX9 marks cortical somatic cells, FN highlights the cortex-medulla border cells (Guioli and Lovell-Badge, 2007) and DMRT1 marks cortical germ cells, not somatic cortical cells (LHX9 positive). In the control ZW female, only the cortical germ cells at the gonad poles are DMRT1 positive (white arrows). In ZZ-E2, ZZ-E2(sr) and ZW-Fa(sr), DMRT1 is expressed in many germ cells across the cortex.

*STRA8* is a major factor involved in meiotic initiation and should be expressed in germ cells of the left cortex in a wave starting from D12 (HH38) (Smith et al., 2008). We analysed the *in situ* expression pattern of *STRA8* in the cortex of ZZ embryos exposed to late oestradiol treatment (ZZ-E2) (Fig. 4A). These were negative for *STRA8* at D14 (HH40) and showed only a few very faint foci at D17 (HH43), compared with the cortex of normal ZW controls, oestradiol-treated ZW embryos (ZW-E2) and ZW embryos exposed to late fadrozole treatment (ZW-Fa) (Fig. 4A).

We also checked the expression of DMRT1, a known key factor involved in the mitotic-meiotic switch in mouse (Matson et al., 2010; Zarkower, 2013). In chick, as in the mouse, DMRT1 is expressed normally in the proliferating germ cells in both differentiating ovary and testis and it is downregulated soon after meiosis starts (Guioli et al., 2014; Omotehara et al., 2014). As shown in Fig. 4Ba,Bc, DMRT1 is downregulated by D17 (HH43) in most germ cells of the wild-type ovary. DMRT1-positive cells were only observed at the poles of the control ovary where the maturation of germ cells is delayed (de Melo Bernardo et al., 2015). In the experimental samples ZZ-E2, ZZ-E2(sr) and ZW-Fa(sr), which were severely compromised for SYCP3 expression, numerous germ cells throughout the cortex were strongly positive for DMRT1 (Fig. 4Bd-Bl). Altogether, these data suggest that it is the presence of testicular tissue that blocks or delays the start of meiosis of the cortical germ cells.

### A cortical domain contributed by ZZ male somatic cells provides a proper niche for ZW and ZZ germ cell meiotic entry

**Fig. 5.**
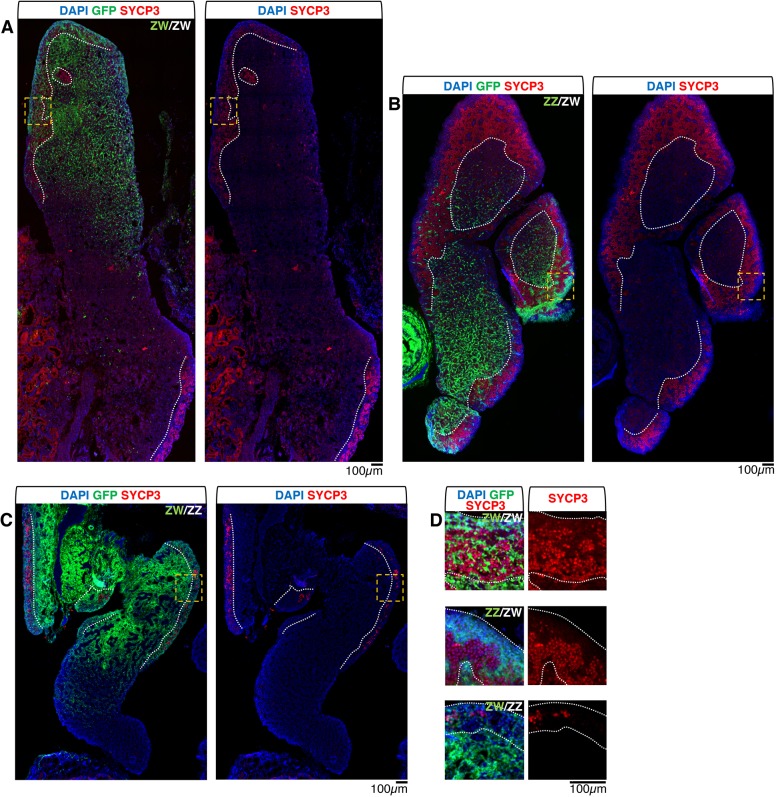
**In mixed-sex gonadal chimeras ZZ somatic cells provide an adequate niche for progression of cortical germ cell into meiosis.** (A-D) Sections from chimeric left gonads at D18 (HH44) immunostained for SYCP3 (red); donor cells are GFP positive (green); nuclei are counterstained with DAPI (blue). (A) ZW donor into ZW host (ZW/ZW) control chimeric ovary; (B) ZZ donor into ZW host (ZZ/ZW); (C) ZW donor into ZZ host (ZW/ZZ); (D) high-magnification images of cortical areas (orange dashed boxes) from A-C (top to bottom, respectively). In ZW/ZW and in ZZ/ZW chimeric left ovaries, most cortical germ cells express SYCP3. In the ZW/ZZ chimeric left ovotestis, SYCP3 is expressed only in some cortical germ cells (see Fig. S3 for visualisation of the distribution of all the germ cells). The ZZ donor cortical somatic cells in the ZZ/ZW chimera provide an adequate niche for progression of germ cells into meiosis. Both ZW and ZZ cortical germ cells can progress into meiosis. However, in the ZW/ZZ ovotestis meiosis is compromised. White dotted line shows the cortex-medulla border.

In order to assess whether a cortical domain made of ZZ somatic cells can sustain the progression of the germ cells into meiosis in physiological conditions, we analysed the expression of the SYCP3 meiotic marker in D18 (HH44) gonadal chimeras (Fig. 5).

In the same-sex female left chimeric ovary (ZW/ZW), most germ cells localised along the entire cortex were positive for SYCP3, as expected (Fig. 5A,D). In ZZ/ZW mixed-sex left chimeric ovary, where the germ cells are female (ZW), the pattern of cortical PGCs was similar to that of the ZW/ZW controls (Fig. 5B,D). This means that SYCP3 was expressed in most PGCs throughout the entire cortex, both in areas mainly contributed by host female (ZW) somatic cells and in areas mostly contributed by donor male (ZZ) somatic cells, demonstrating that the latter are competent to form a proper cortical germ cell (oocyte) niche. However, in ZW/ZZ left chimeric ovotestis the results were more complex. Within the ovarian portion the cortical germ cells showed a variable pattern depending on localisation. In some cortical areas, many germ cells were positive for SYCP3, whereas in other areas few or no germ cells were positive for these markers (Fig. 5C,D). The fact that at least some ZZ germ cells enter meiosis in this ZW/ZZ chimera, indicates that both female and male germ cell chromosomal sex identity is compatible with starting the female meiotic programme in the embryo. However, the SYCP3 patchy pattern suggests that the ovotestis condition of the chimera (see Fig. S3 for the chimera structure) may generate a ‘confused’ sex environment that blocks or severely delays meiotic entry, similar to what was observed in the ZZ-E2, ZZ-E2(sr) and ZW-Fa(sr) models.

### ERα downregulation in the ZW left gonad epithelium disrupts cortex differentiation

At sex determination, *ERα* is asymmetrically expressed in both ZW and ZZ embryos, being restricted to the epithelium of the left gonad although present in both medullas (Andrews et al., 1997; Guioli et al., 2014). This pattern makes it a candidate oestrogen signal transducer in promoting cortical differentiation. In this study, we showed that the ZZ left gonad is sensitive to oestrogen beyond the initiation of male-specific differentiation, implying that an oestrogen transducer should be expressed asymmetrically in the differentiating testis. However, this had not been specifically investigated in previous studies. To address this issue, we performed a time-course analysis of ERα during testis differentiation by immunostaining. ERα expression was clearly detected in the epithelium of the left ZZ gonad at D7 (HH31). Although weaker than in the differentiating ovary, the staining was maintained in the epithelium of the left testis until at least D12 (Fig. S5). This indicates that the left epithelium of the ZZ developing testis retains the potential to respond quickly to oestrogen, even though it would not normally be exposed to it at any significant level.

**Fig. 6.**
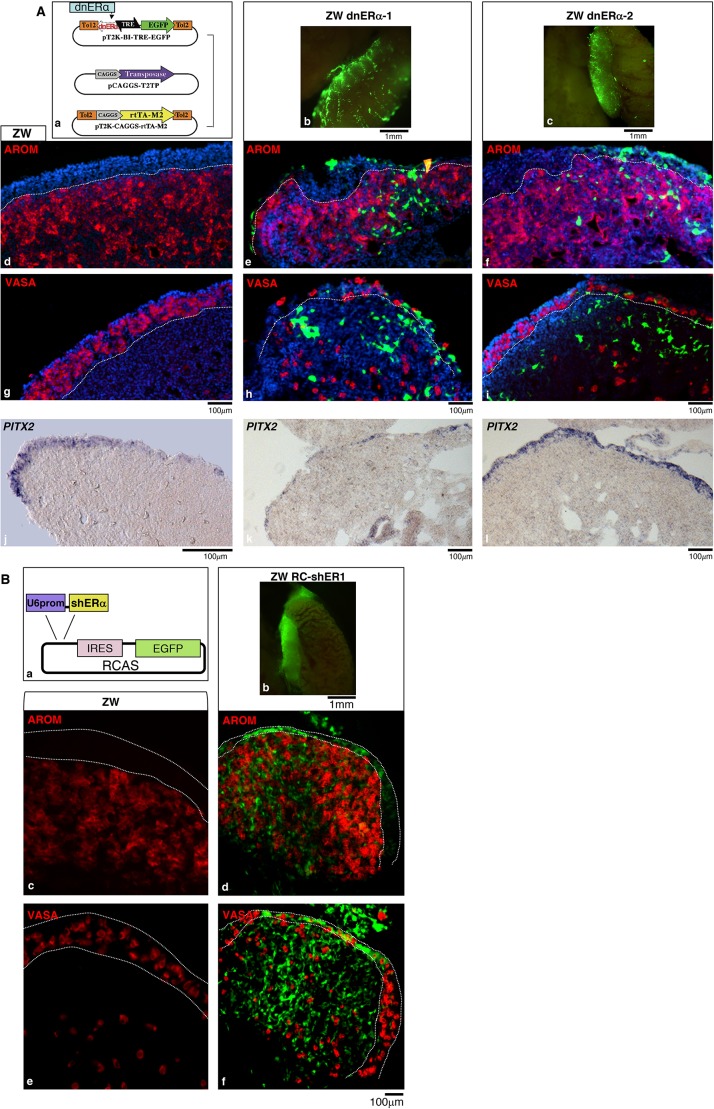
***In*** ***ovo* suppression of epithelial ERα activity disrupts cortex differentiation.** (A) Electroporation of a dominant-negative isoform of *ERα* (dnERα) to the gonadal epithelium. (Aa) Schematic of the inducible TET-ON plasmid system used to express dnERα (see Materials and Methods for details), LBD, ligand binding domain. (Ab,Ac) Whole-mount images of two ZW left gonads electroporated at D2.5 (HH15-17) and screened at D10 (HH36); targeted cells are highlighted by expression of an EGFP reporter (green). (Ad-Ai) Fluorescence images of D10 (HH36) gonad sections immunostained for P450 aromatase (AROM; red), or the germ cell marker VASA (red) and GFP (green); (see Fig. S7 for the EGFP pattern alone) from ZW control (Ad,Ag) and ZW electroporated (Ae,Af,Ah,Al) gonads. (Aj-Al) *PITX2* RNA *in situ* expression pattern in ZW control (Aj) and ZW electroporated (Ak,Al) gonads. White dotted lines show the cortex/medulla border. (B) Electroporation of RCAS retroviral DNA expressing *ERα* shRNA (RCAS-shER1) to the gonadal epithelium. (Ba) Schematic of the construct: RCAS-shERα expressing *ERα*-specific short hairpin RNA molecules in tandem with an EGFP reporter. (Bb) Whole-mount image of a ZW left gonad electroporated with RC-shER1 at D2.5 (HH15-17) and screened at embryonic D9 (HH35-36) based on the EGFP reporter expression. (Bc-Bf) Fluorescence images of D9 (HH35-36) gonad sections immunostained for P450 aromatase (AROM; red) or the germ cell marker VASA (red) (see Fig. S7 for the EGFP pattern alone) from ZW control (Bc,Be) and ZW-RCAS-shER1 (Bd,Bf) gonads. The cortex is compromised by downregulation of ERα activity in both model systems compared with controls. Four out of eight samples from the two experiments showed the severe cortical phenotype in well-electroporated regions. See Fig. S8 for immunofluorescence results on sections from electroporation of various control vectors expressing an EGFP reporter (minimum of three replicates per control vector analysed).

In order to knock down the activity of ERα in the epithelial cells of the left ovary, a dominant-negative form of *ERα* (*cERα524*) (see Fig. S6A for assessment of dnERα activity) cloned into a Tet-ON plasmid for conditional gene expression, was transfected into the left gonadal epithelium at D2.5 (HH15-17) by *in ovo* electroporation of dorsal coelomic epithelium on the left side (Guioli et al., 2007). The DNA was co-electroporated with other plasmids of the Tet-ON system, including T2TP for expression of the Tol2 transposase and pT2K-CAG-rTA-M2, the doxycycline-inducible activator (Watanabe et al., 2007) (Fig. 6Aa; see Materials and Methods). Expression of *cERα524* was induced at D4.5 (HH25) or D5.5 (HH27) and the screening was carried out at D10 (HH36) based on the EGFP reporter expression, as a measure of the quality of transfection.

Electroporation typically results in mosaic transfection that varies from sample to sample. ZW embryos displaying high levels of EGFP (e.g. Fig. 6Ab,Ac) were processed for immunostaining with markers specific to different cell types. The most severe phenotype was observed in ovaries exposed to doxycycline at the earliest time point. An example is shown in Fig. 6Ab,Ae,Ah,Ak. In the posterior part of the ovary, most of the medulla highlighted by P450 aromatase was overlain by a simple epithelium, with the exception of the central part, which displayed some stratification and contained a small aggregate of germ cells (Fig. 6Ae,Ah) (see Fig. S7 for better visualisation of the GFP pattern). A milder phenotype is shown in Fig. 6Ac,Af,Ai,Al where the cortex appears markedly thin in areas where stretches of the epithelium were still enriched in EGFP compared with the non-targeted portion. In these areas few germ cells were present (Fig. 6Ai). Although by D10 (HH36) many EGFP-positive epithelial-derived cells were found in the medulla, the medulla maintained a female identity, being positive for P450 aromatase (Fig. 6Ae,Af) and negative for SOX9 (Fig. S8). No other obvious phenotype was observed in the medulla. All samples still expressed *Pitx2* within the left epithelium as seen in the normal controls (Fig. 6Aj-Al).

To make sure that the data observed were due to specific knockdown of ERα activity, we performed an alternative set of experiments aiming to suppress ERα via RNAi. Fig. 6B shows a left ovary screened at D9 (HH35-36) and analysed for germ cell localisation and cortical structure. It is an example of a left gonad very well-targeted in large areas along the anterior/posterior axis. As with the most severe cERα524 phenotype, the ERα shRNA-targeted left gonad showed a female medulla similar to the ZW wild-type control, but a severely compromised cortex, corresponding with well-targeted areas of the epithelium. The medulla, marked by P450 aromatase was found to be extended up to the base of the epithelium in the middle portion of the ovary, where most epithelial cells were EGFP positive, indicating lack of cortical development in this region (Fig. 6Bd). The absence of a stratified cortex at D9-10 was never observed in ZW wild-type gonads (see controls in Fig. 6A,B) and in gonads electroporated with various control vectors expressing an EGFP reporter, even in areas that were very well transfected (Fig. S9).

## DISCUSSION

In recent years, great advances have been made uncovering the key sex regulators that control the commitment to the male and female programmes, mainly based on work carried out in mammals, primarily in the mouse.

However, ovarian differentiation is still poorly understood. One of the main reasons is that mouse ovarian morphogenetic changes, including the formation of a cortex, are delayed to the perinatal period, making the discovery of female sex-promoting factors more challenging. Moreover, oestrogen is dispensable for mouse embryonic ovary differentiation, maybe reflecting the evolution of a different hierarchy of early regulators, compared even to other mammals. One example of this is *Foxl2*, which is crucial for ovary differentiation early in gonadal development in the goat, but not until after birth in the mouse (Boulanger et al., 2014; Schmidt et al., 2004; Uda et al., 2004; Uhlenhaut et al., 2009).

The chicken ZW gonad produces oestrogen and differentiates into an ovary with a clear cortex and medulla soon after the female molecular pathway initiates, making it a good model system in which to address early ovarian differentiation. In this study, we focused on cortical morphogenesis with the aim of understanding the differentiation of the female (i.e. oogenic) germ cell niche.

Oestrogen signalling is an important factor in chicken female sex determination, as it can override the genetic sex if perturbed at the time of sex determination (Yang et al., 2008). This holds true in lower vertebrates and some mammals, including marsupials (Coveney et al., 2001; Guioli et al., 2014). However, its potential roles as anti-testis and ovarian-promoting factor are still unclear. To explore its action in promoting cortex formation and to understand if this process can be dissociated from female specification of the medulla, we observed the effect of manipulating oestrogen levels after D7 (HH31), when sex reversal is no longer achievable and medulla differentiation is consistent with genetic sex. Moreover, as the fate of the medulla, in chicken, is determined by a cell-autonomous process strictly based on sex chromosome identity, we also generated a series of male:female gonadal chimeras to assess whether CASI holds true for the cortical domain.

The analysis of same-sex and mixed-sex left gonadal chimeras showed that ZZ and ZW cells can become part of a cortex that can provide a proper oogenic niche like the female counterpart. In both cases, after an initial phase of proliferation, the germ cells entered meiosis at the appropriate time, excluding the involvement of W- or Z-specific genes in the control of early cortical development. Therefore, the cortex does not form based on the CASI process that determines the medullary fate (Zhao et al., 2010). It is known that the Z gene *DMRT1*, a current candidate as a male primary sex determinant in chicken (Guioli et al., 2014; Lambeth et al., 2014; Smith et al., 2009), is expressed symmetrically in the medulla, but also asymmetrically in the gonad epithelium of ZZ and ZW embryos at the time of sex determination (Guioli and Lovell-Badge, 2007; Omotehara et al., 2014). As *DMRT1* is not subject to dosage compensation (McQueen and Clinton, 2009), our chimeras show that any differences in *DMRT1* expression levels due to gene dosage between female and male somatic cells does not impact cortex formation.

The oestrogen injection in D7 ZZ embryos generated an ‘intersex’ gonad composed of a male medulla overlain by a female cortex, whereas downregulation of oestrogen in ZW embryos perturbed proper cortex formation, maintaining a female medulla. These results show that the cortex domain can, at least initially, differentiate independently from the sex of the medulla if oestrogen is provided. This makes oestrogen the major promoting signal for cortex development and the left epithelium a naïve tissue capable of responding to any environmental sex hormone influence.

Therefore, early cortex development depends on a non-cell-autonomous process triggered by sex-promoting signals, notably oestrogen, normally produced in the ovarian medulla. Consistent with our results, a previous study inhibiting oestrogen synthesis in *Trachemys scripta* at female producing temperature has shown that cortical expansion does not require a feminised medulla in *T. scripta*, supporting the idea that the differentiation of medulla and cortex may be uncoupled, at least initially (Barske and Capel, 2010).

However, we did observe that in the left gonad of ZZ embryos subject to late oestradiol treatment and in ovotestes commonly obtained from ZW/ZZ chimeras, cortical germ cell progression into meiosis was compromised, as SYCP3, a marker of the synaptic process was absent or expressed in very few cells at D17-18 of embryogenesis. Although in these severely compromised models the PGCs were genotypically male, there is evidence that this failure is not a result of the germ cell genotype. For example, although meiosis was compromised in ZW/ZZ chimeras, patches of SYCP3-positive ZZ germ cells were found, suggesting that the ZZ germ cells were capable of initiating meiosis at the right time in some areas of the cortical domain. Moreover, left ovotestes, generated by manipulation of oestrogen levels in either D4 ZZ or ZW embryos, both contained cortical germ cells with compromised SYCP3 expression. This therefore occurs independently of the germ cell chromosomal sex.

In chicken, as in the mouse, *STRA8* is a key factor for germ cell entry into meiosis (Bowles et al., 2016; Koubova et al., 2014; Smith et al., 2008; Yu et al., 2013). In our SYCP3 compromised models *STRA8* was almost undetectable throughout development, whereas DMRT1, which should be downregulated at meiosis, was still present in many D17 (HH43) germ cells. This shows that at D17 (HH43) these cortical domains contained germ cells that have not progressed properly into meiosis, suggesting meiotic arrest or at least a severe delay.

The mitotic-meiotic switch is a very complex process, including many germ cell intrinsic and extrinsic factors that may act as promoters or suppressors of meiosis, but which are not yet completely elucidated. Our results suggest that signalling originating outside the cortex must be involved. It has been shown that FGF9 secreted by the Sertoli cells antagonises meiotic entry via activation of Nodal-Smad signalling in mouse spermatogonia (Tassinari et al., 2015) and in chicken ovary bFGF was found to act as a suppressor of meiosis and as a mitogenic factor (He et al., 2012). The male medulla in the ovotestes may antagonise meiotic entry via FGF signalling or other male-driven signalling. Alternatively, meiosis may be impaired by the lack or deficiency of some female inducer signals from the medulla, or both.

In humans, disorders of sexual development are associated with an increased risk of type II germ cell cancer, a form of cancer derived from two types of *in situ* neoplastic lesions: carcinoma *in situ* (CIS) or gonadoblastoma, depending on the supporting cells being Sertoli cells or granulosa cells, respectively (Hersmus et al., 2017). The observation of mitotic and meiotic signals within CIS cells, together with the inability of these cells to ever enter meiosis, points to a dysfunctional mitotic:meiotic switch that could provide the genetic instability initiating the tumour (Cools et al., 2006; Jorgensen et al., 2013). The long-standing hypothesis is that this process is initiated in fetal life, where a gonad with an intersex phenotype provides a sexually confused niche, which can result in the delay or arrest of germ cell development. If these cells are not eliminated they may retain the embryonic phenotype and later transform (Jorgensen et al., 2013).

These human studies support the idea that the meiotic phenotype in our intersex chick models is due to the sexually confused environment and predict that if these germ cells are not later eliminated, they could transform into tumour cells. The chicken is already used as a model of ovarian cancer as it develops spontaneous ovarian cancer at a rate similar to human (Bernardo et al., 2015). The oestrogen-manipulated chick models might provide an important system in which to study the origin and dynamics of neoplastic transformation of germ cells.

We have previously proposed that oestrogen signalling could be transduced via ERα on the basis of the RNA expression pattern at sex determination (Andrews et al., 1997; Guioli and Lovell-Badge, 2007). The symmetric expression in left and right medulla and the asymmetric expression in the gonad left epithelium of both female and male embryos suggested that oestrogen, via ERα, may have multiple roles in promoting ovary differentiation affecting the differentiation of both the medulla and the cortex. However, the protein pattern in the male has always remained elusive, making it difficult to explain how radiolabelled oestradiol injected in the embryo found to be binding both female and male gonad at D5-10 may do so via ERα (Gasc, 1980). In the present study, we were able to show that some levels of ERα protein are present in the male gonad at sex determination in a pattern that reflects the RNA expression, indicating that both ZW and ZZ gonad could quickly respond via ERα to oestrogen stimulation. Moreover, the maintenance of expression within the left epithelium in the differentiating testis shows that the epithelium remains exquisitely sensitive to oestrogen and able to respond rapidly to the hormone even several days into the male differentiation pathway.

In agreement with predictions, the knockdown of ERα activity in the epithelial cells of the left ZW gonad has resulted in the impairment of cortical development, the most severe cases displaying areas devoid of a cortex and overlain by a simple epithelium, similar to the phenotype observed in the D10 embryos treated with fadrozole from D7-7.5 (HH31). The downregulation only affected the cortical domain without affecting the sex identity of the medulla. Moreover, the cortical phenotype was not linked to variation in *PITX2* expression within the left epithelium. Altogether, these data indicate that ERα is the main transducer of oestrogen signalling in cortex formation and that this process strictly depends on its activity within the left epithelial cells of the gonad.

ER is a complex molecule. It has been shown in different systems that, upon oestrogen activation, it can have non-genomic and genomic activities. It can rapidly respond at the plasma membrane by triggering signalling pathways and it can mediate transcriptional regulation within the nucleus (Hamilton et al., 2017). Our future work will address the mechanism of action of ERα in promoting cortical development upon oestrogen stimulation, through the identification of the downstream targets of its action via transcriptomics approaches.

In summary, our data suggest a model whereby the gonads, at the time of somatic sex-specific differentiation, are formed of two distinct domains: the epithelium and the medulla. Left and right medulla differentiates along the male or female pathway strictly based on CASI, depending on whether the cells within it are ZW or ZZ. CASI does not apply to the formation of the cortex. The epithelium of the left gonad is naïve with respect to chromosomal sex identity. If exposed to oestrogen it will develop a cortex, via activation of ERα. The finding that this activation can occur above a male medulla, shows that oestrogen is sufficient for this step and suggests that the initial formation of the cortex relies on positive inductive signals rather than the lack of antagonising signals from the medulla.

Later progression of the cortical germ cells into meiosis requires a medulla of the correct phenotypic sex, suggesting that meiotic entry is a checkpoint of a well-coordinated ovarian cortex/medulla differentiation pathway.

Our results provide new insights into the process of chicken ovarian differentiation and provide a model that could be extended to other systems, including many mammals. Moreover, our data suggest that the chick intersex models may be a potential valuable system for investigating the aetiology of germ cell tumours.

## MATERIALS AND METHODS

### Animals

Most experiments were performed using Dekalb White chicken eggs obtained from Henry Stewart, Louth, UK, except for the generation of chimeras, which was carried out using GFP transgenic chickens (Roslin Greens) and ISA Brown chickens held at the National Avian Research Facility (NARF) at the Roslin Institute, Edinburgh, UK. All experiments carried out on chickens were performed in accordance with regulations of the Francis Crick and Roslin Institute's Animal Welfare and Ethical Review Body (AWERB), under the UK Animal (Scientific Procedures) Act.

### Plasmids and *in ovo* electroporation

The Tol2 integration plasmid system was a kind gift of Dr Yoshiko Takahashi (Kyoto, Japan) and includes: the transposase expression plasmid pCAGGS-T2TP, the expression plasmid pT2K-CAGGS-EGFP and the conditional expression plasmid PT2K-B1-TRE-EGFP (both carrying an EGFP reporter), and the Tet-ON activator plasmid pT2K-CAG-rTA-M2, which responds to doxycycline (Sato et al., 2007; Watanabe et al., 2007). A truncated isoform of *ERα* lacking the last 196 bp of the open reading frame (last 65 amino acids of the ligand-binding domain) was generated by PCR using primers F-5′ACTAGTTCTAGAGCCATTAGCAATGACCATGAC and R-5′GTCGACTCTAGATTAACACTTCATATTGTACAGGTG, cloned in pCRII-TOPO (Invitrogen) and then moved into PT2K-B1-TRE-EGFP (using BamHI and EcoRV sites) to make *cERα524*. This isoform corresponds to a human *ERα* isoform shown to have a powerful dominant-negative activity (Ince et al., 1993).

Three *ERα* shRNA molecules were cloned downstream of a mouse U6 promoter into pCRIITOPO using a PCR-based approach aiming to amplify the U6 promoter along with the shRNA, similar to the approach described by Harper et al. (2005). Common forward primer: U6-Forward, 5′TCTAGATCGACGCCGCCATCTCTAG; U6-reverse-shRNA specific primers:

(ER1) CTCGAGAAAAAAGGAACACCCAGGAAAGCTTTCCCATCTGTGGCTTTACAGAAAGCTTTCCTGGGTGTTCCAAACAAGGCTTTTCTCCAAGGG;

(ER2) CTCGAGAAAAAAGCCACTAACCAGTGTACTATCCCATCTGTGGCTTTACAGATAGTACACTGGTTAGTGGCAAACAAGGCTTTTCTCCAAGGG;

(ER3) CTCGAGAAAAAAGGTACCCTACTACCTTGAAACCCATCTGTGGCTTTACAGTTTCAAGGTAGTAGGGTACCAAACAAGGCTTTTCTCClAAGGG.

The sequences include restriction enzymes for cloning. Dominant-negative activity of cERα524 was confirmed by luciferase assays *in vitro*, following transfection in HEK293 cells (ATCC-CRL-3216) together with full length *cERα* (cloned into pSG1) and a 3XERE-TATA-Luc luciferase reporter (Lemmen et al., 2004) (kind gift of Dr Paul T. van der Saag, Hubrecht Institute, Utrecht, The Netherlands); the three shER RNA molecules were also tested by luciferase assay using the same *in vitro* system; three biological replicates were performed in triplicate (see Fig. S6 for details and result). U6-shER ER1, displaying the best silencing activity was cloned into pSLAX13, previously modified to carry IRES-EGFP. U6-shER-IRES-EGFP (ER1) was then moved to a RCAS(A) retroviral vector to generate RCAS-U6-shER-IRES-EGFP (RCAS-shER1).

*In ovo* electroporation of plasmid DNA into the left gonadal epithelium was achieved by DNA injection into the left coelomic cavity of HH15-17 embryos using a glass capillary needle and an Inject+matic pico pump, followed by electroporation using a NEPA21 electroporator (Sonidel) (five 50 ms pulses at 26 V, transferring pulse only). The detailed procedure was previously described (Guioli et al., 2007). Experiments were repeated more than three times using at least 40 eggs per experiment. The surviving rate at collection was around 30%. The dissected gonads were screened on the basis of the EGFP expression pattern in the gonads. A minimum of four of the best electroporated gonads, per type, and wild-type gonads collected at D10 were analysed. See Fig. S8 for details of electroporation of other control vectors.

### *In ovo* drug treatment

For manipulation of oestrogen levels after sex determination (late treatment), eggs were incubated at 37.7°C pointed end down. At D7-7.5 (HH31) or D9 (HH35) a first injection in the air chamber was performed through a hole made at the rounded end. To downregulate oestrogen, the P450 aromatase inhibitor fadrozole (Sigma-Aldrich, F3806) was injected at 0.5 mg/egg in 50 μl of PBS and then re-injected at 0.3 mg/egg every other day. To increase oestrogen, β-oestradiol (Sigma-Aldrich, E2758) was injected at 120 µg/egg in 25 µl of 95% ethanol; embryos collected at D17 (HH43) were injected once more at D13 (HH39). For manipulation of oestrogen levels before sex determination (early treatment) eggs were injected once at D4 (HH23) with 0.5 mg or 1 mg/egg of fadrozole, or 120 µg/egg of β-oestradiol. Three or more gonadal pairs were analysed from each type of experimental treatment, per developmental stage. A minimum of two stages were assessed (D10 and D17).

### Generation of gonadal chimera

D2 (HH10/12) (13-15 somites) GFP transgenic embryos (Roslin Greens) and ISA Brown embryos were used as donor and host, respectively. Details of the procedure have been described by Zhao et al. (2010). The manipulated host embryos were then incubated until D18 (HH44). Two or three well-contributed same sex or mixed sex gonadal chimeras per type were analysed further at D18.

### Antibodies, immunohistochemistry and *in situ* hybridisation

The following antibodies were used: rat anti-VASA (1:1000) (Aramaki et al., 2009), rat anti ERα (1:200, Fitzgerald, 10R-E124AX), rabbit anti-DMRT1 (1:1500) (Guioli and Lovell-Badge, 2007), goat anti-SOX9 (1:500, R&D Systems, AF3075), goat anti-FOXL2 (1:400, Abcam, ab5096), mouse anti-P450 aromatase (1:200, Serotec, MCA2077S), mouse anti-γH2AX (1:200, Merck, 05-636), rabbit anti-SYCP3 (1:500, Novus Biologicals, NB300-232), mouse anti-fibronectin (1:100, Developmental Studies Hybridoma Bank, B3/D6), goat anti-LHX9 (1:200, Santa Cruz Biotechnology, sc-19350). The *STRA8* probe template for RNA *in situ* was generated by PCR cloning into PCRIITOPO (Invitrogen), using primers F-5′TACCCAGACACCTCATCCCC and R-5′TCAAAGGTCTCCGTGCACCG. The *PITX2* probe was previously described (Logan et al., 1998). Urogenital ridges were fixed in 4% paraformaldehyde at 4°C overnight, rinsed in PBS at room temperature, then transferred to 30% sucrose overnight and finally embedded in OCT and stored at −80°C. Cryosections for *in situ* hybridisation were processed based on the protocol described by Moret et al. (2004). Cryosections for immunofluorescence were rinsed three times (5 min each) in PBS and transferred for 1 h to a blocking solution (PBS/0.1% Triton X-100, 2% donkey serum) before incubating with the primary antibody overnight at 4°C (or 37°C for ERα). After three 10 min washes in PBS/0.1% Tween 20, the sections were incubated with secondary antibodies (Alexa Fluor-conjugated donkey antibodies, Invitrogen, A10037, A21206, A21209, A10042, A21202, A11055, A31571, A21447; 1:400 in PBS/0.1% Tween 20) for 1 h at room temperature. The results from the *in situ* hybridisation on cryosections were imaged using a Leica DM-RA2 upright microscope equipped with a Retiga 200R camera and Q-capture Pro7 software (Q imaging). Fluorescence images were collected on an Olympus VS120 slide scanner equipped with an XM10 monochrome camera and a VS-ASW-L100 software (Olympus), or on a Leica upright SPE confocal system.

## Supplementary Material

Supplementary information
