# Supplementary material for: Oestrogen in the chick embryo can induce chromosomally male ZZ left gonad epithelial cells to form an ovarian cortex that can support oogenesis
Source: Development. 2020 Feb 25;147(4):dev181693. doi: 10.1242/dev.181693 (PMC7055392; doi:10.1242/dev.181693)
Supplement: Supplementary information [file develop-147-181693-s1.pdf]

Fig. S1

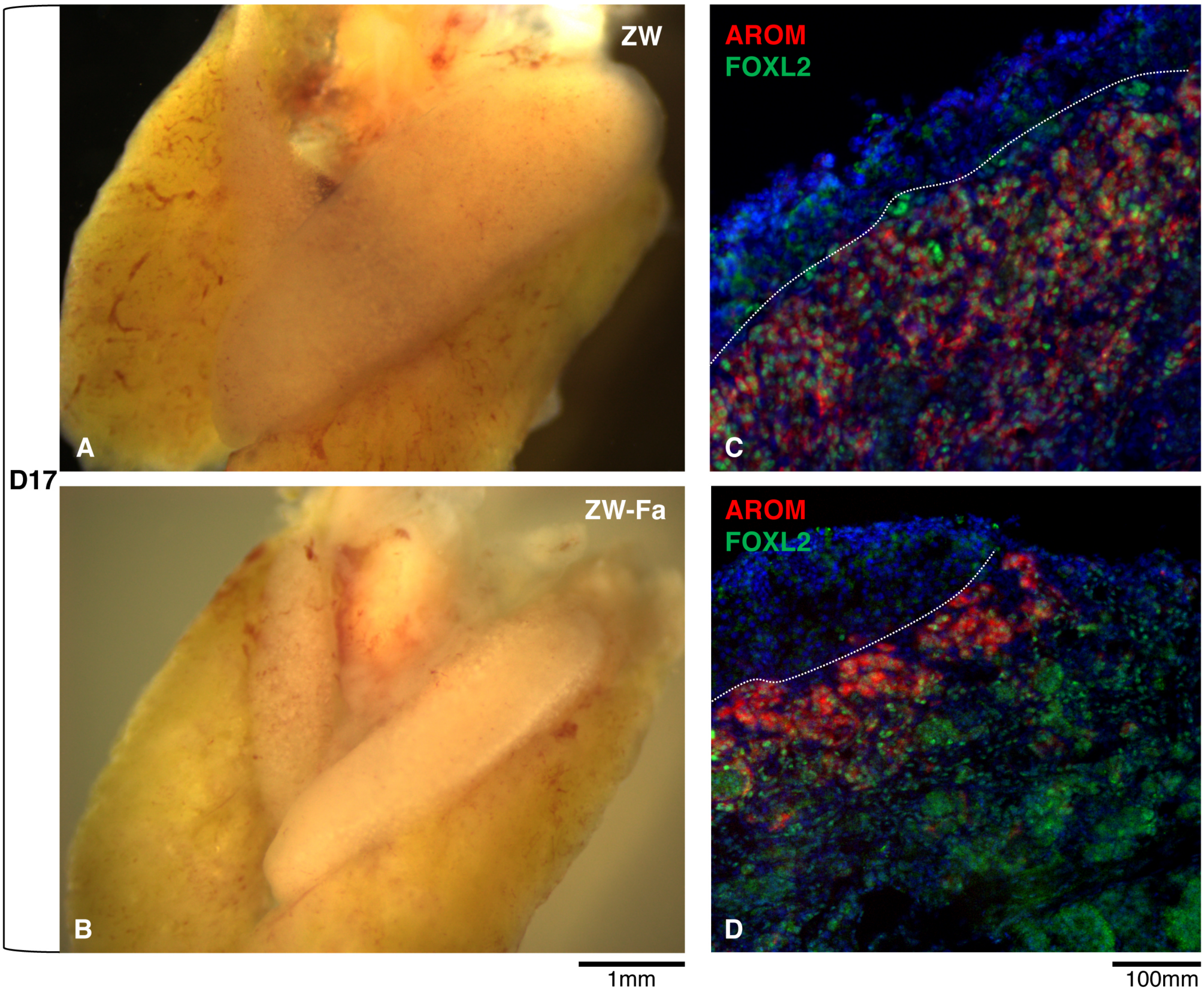

Fig. S1 In ZW embryos treated with Fadrozole at D7-7.5 (HH31) the growth of the left ovary is affected. (A-B) D17 (HH43) ZW gonads, (A) ZW control, (B) ZW treated with Fadrozole from D7-7.5 (ZW-Fa); (C, D) fluorescence images of cryostat sections from the left gonad in (A) and (B) respectively, stained for the female markers P450aromatase (AROM; red) and FOXL2 (green); nuclei counterstained with DAPI (blue). White dotted lines highlight the cortex-medulla border.

Fig. S2

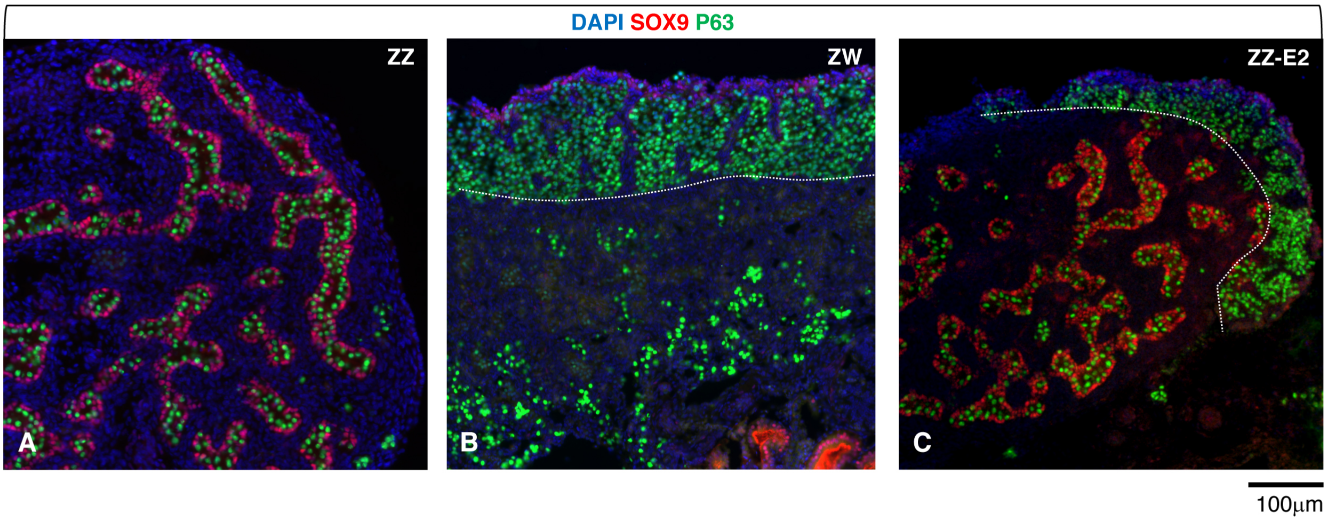

Fig. S2 The left testis of ZZ embryos exposed to ß-estradiol at D9 (HH35) develops a cortical domain. Sections from the left gonad of D17 (HH43) embryos, immunostained for the Sertoli cell marker SOX9 (red) and the germ cell marker P63 (green); nuclei counterstained with DAPI (blue). (A) ZZ control, (B) ZW control, (C) ZZ treated with ß-estradiol (ZZ-E2) at D9 (HH35). White dotted lines highlight the cortex-medulla border.

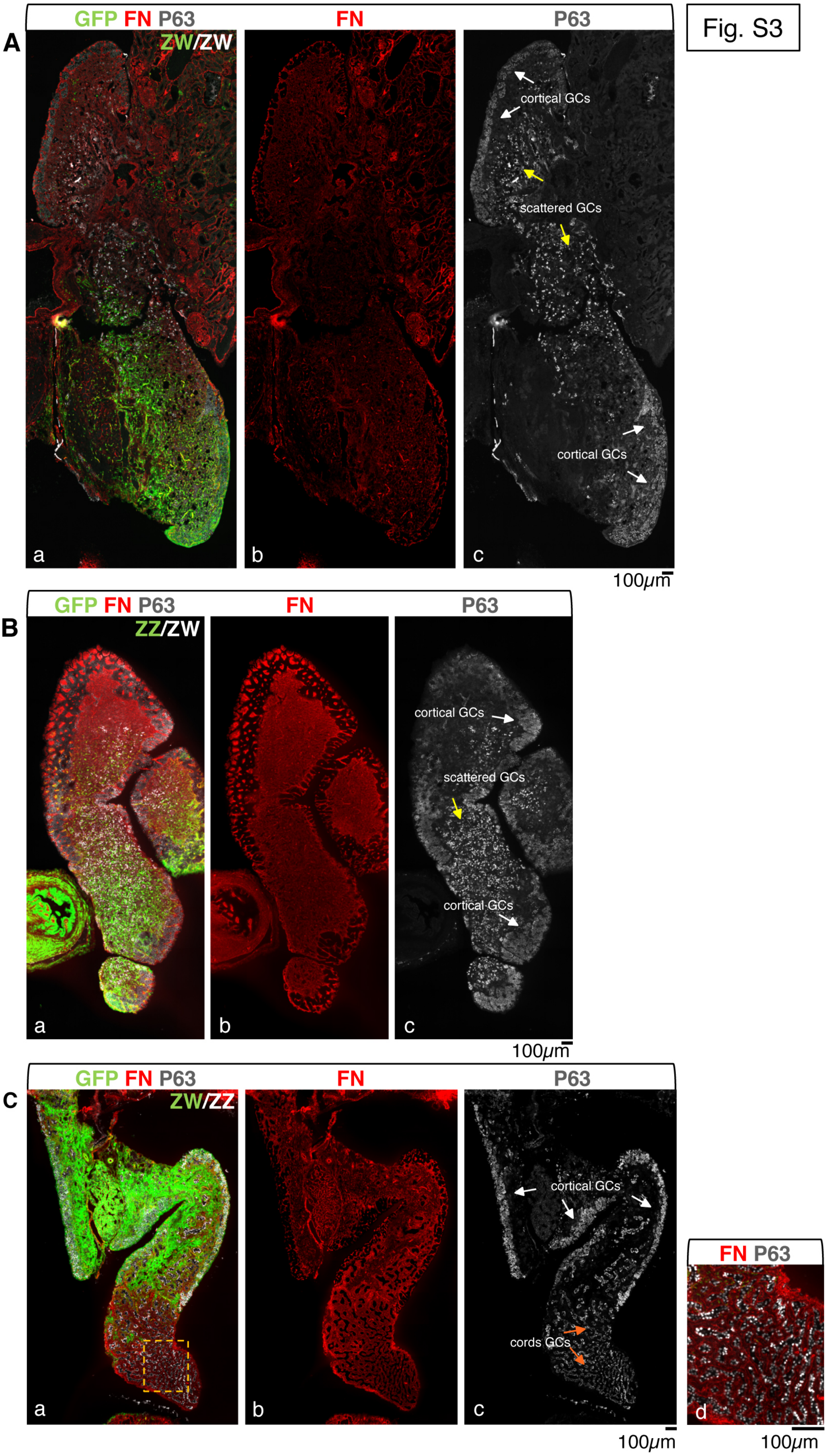

Fig. S3
A
GFP FN P63
ZW/ZW
FN
P63
cortical GCs
scattered GCs
cortical GCs
a
b
c
100μm
B
GFP FN P63
ZZ/ZW
FN
P63
cortical GCs
scattered GCs
cortical GCs
a
b
c
100μm
C
GFP FN P63
ZW/ZZ
FN
P63
cortical GCs
cords GCs
a
b
c
100μm
FN P63
d
100μm

Fig. S3 ZW and ZZ cells can both contribute to the somatic component of the cortex. Immunofluorescent images of sections from the D18 (H44) left chimeric gonads shown in Fig. 2, displaying germ cells localisation using the marker P63 (grey), the structure of the gonad using the extracellular matrix (ECM) marker Fibronectin (FN; red) and the GFP positive donor cells (green). (A) ZW donor into ZW host (ZW/ZW); (B) ZZ donor into ZW host (ZZ/ZW); (C) ZW donor into ZZ host (ZW/ZZ). Combined FN/P63 (Aa,Ba,Ca) and individual FN (Ab,Bb,Cb) and P63 (Ac,Bc,Cc) stainings are shown. White and yellow arrows indicate cortical and medullary scattered germ cells (GCs), respectively. Orange arrows indicate GCs within testicular cords of the ovotestis. Details of ZW/ZZ chimeric ovotestis cords in orange box (Ca) shown in panel (Cd).

Development • Supplementary information

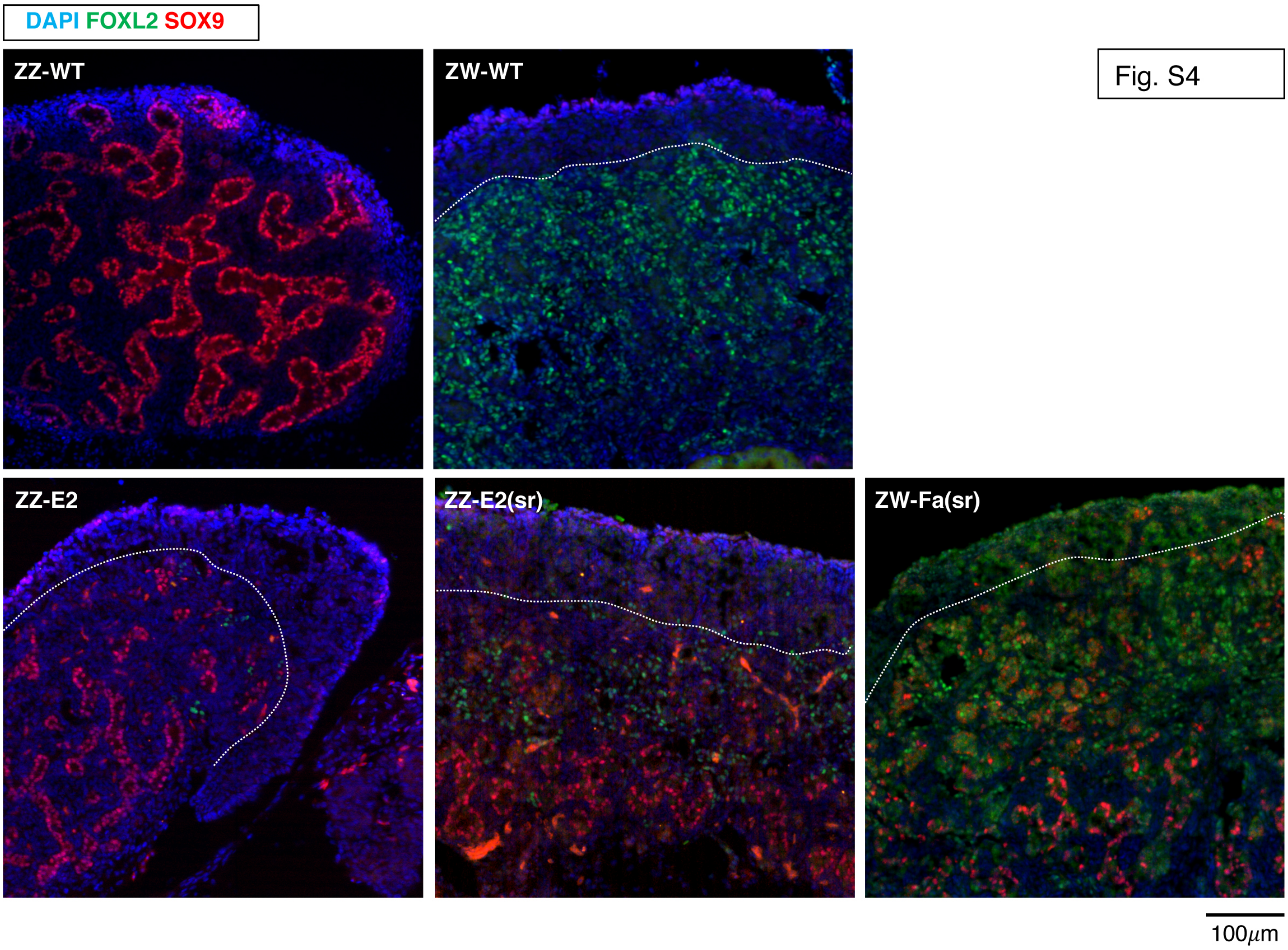

Fig. S4 Phenotypic sex of the gonadal medulla in D17 embryos subject to estrogen levels alteration. D17 (HH43) left gonad sections immunostained for the medulla female specific marker FOXL2 (green) and male specific marker SOX9 (red); nuclei are counterstained with DAPI (blue). ZZ-WT, ZZ wildtype; ZW-WT, ZW wildtype; ZZ-E2, ZZ treated with ß-estradiol at D7-7.5; ZW-Fa(sr), ZW, partially sex reversed gonad, from treatment with Fadrozole at D4; ZZ-E2(sr), ZZ, partially sex reversed gonad, from treatment with ß-estradiol at D4. White dotted lines highlight the cortex-medulla border.

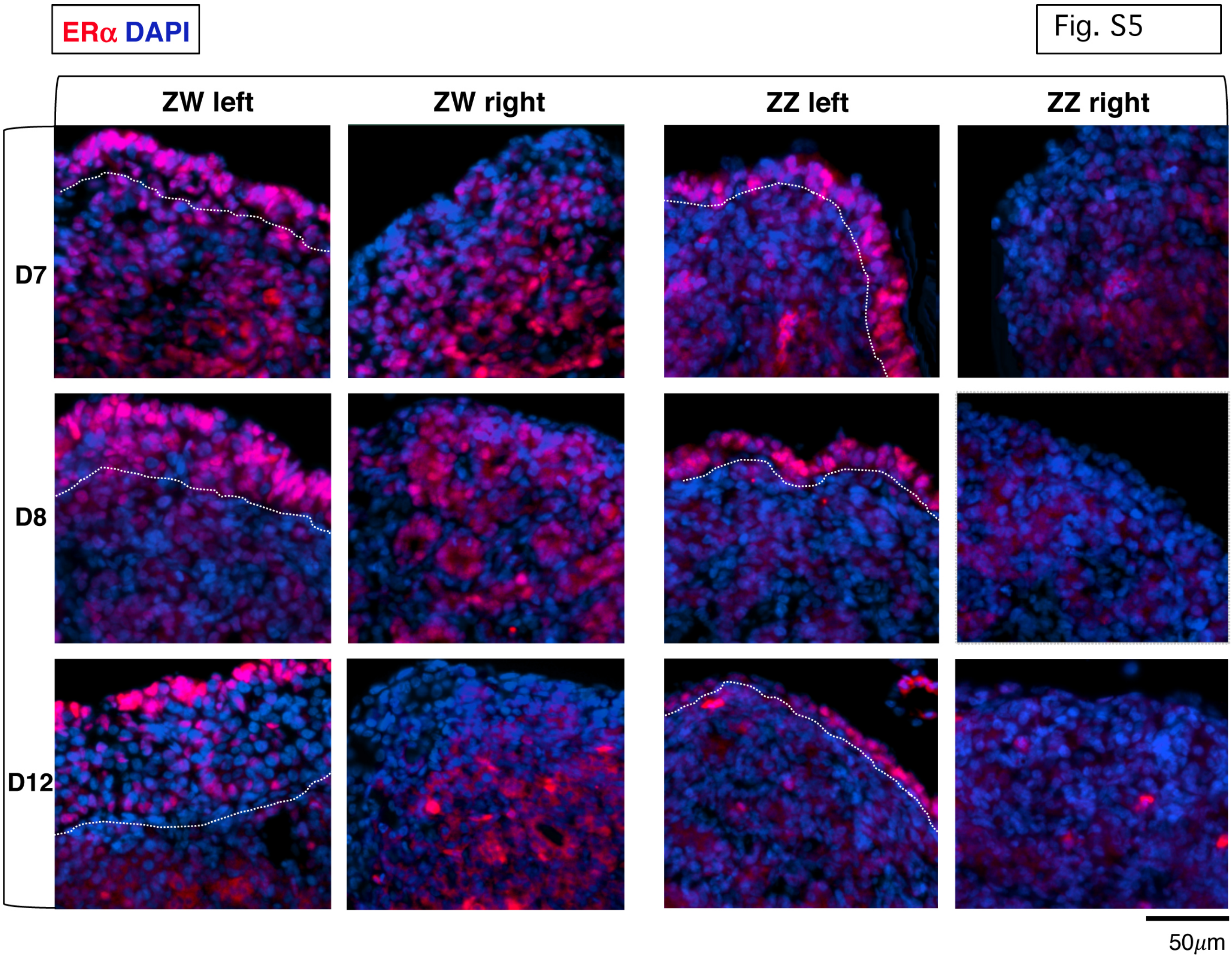

Fig. S5 ERα protein is produced in the epithelium of the left ZZ gonad during differentiation. Sections from ZW and ZZ left and right wild type gonads at embryonic D7 (HH31), D8 (HH34) and D12 (HH38), immunostained for ERα (red). Nuclei are counterstained with DAPI (blue). White dotted lines highlight the cortex or epithelium-medulla border.

Fig. S6

A

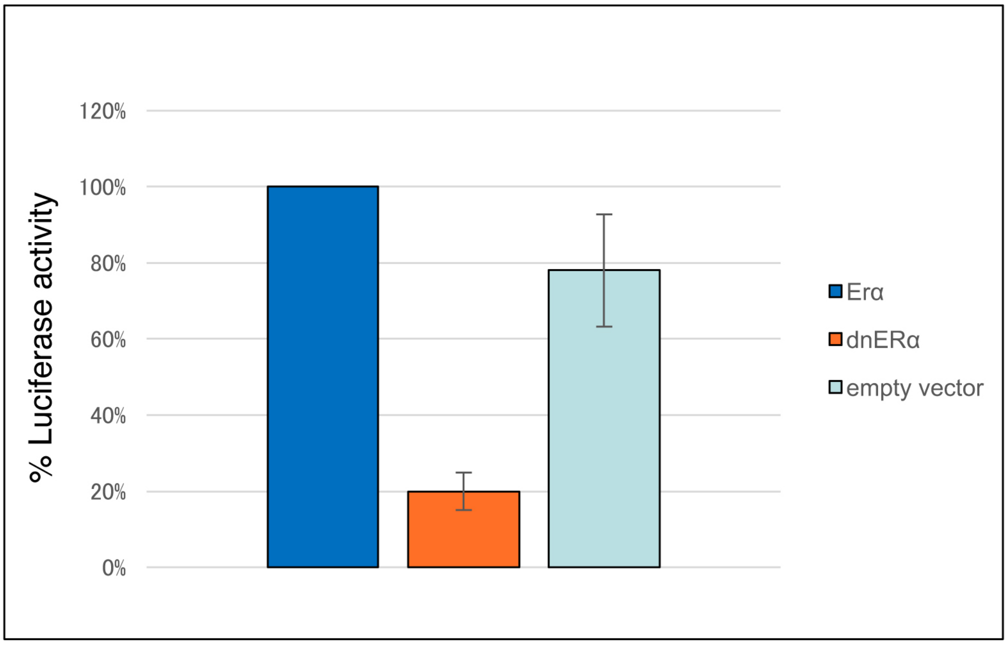

B

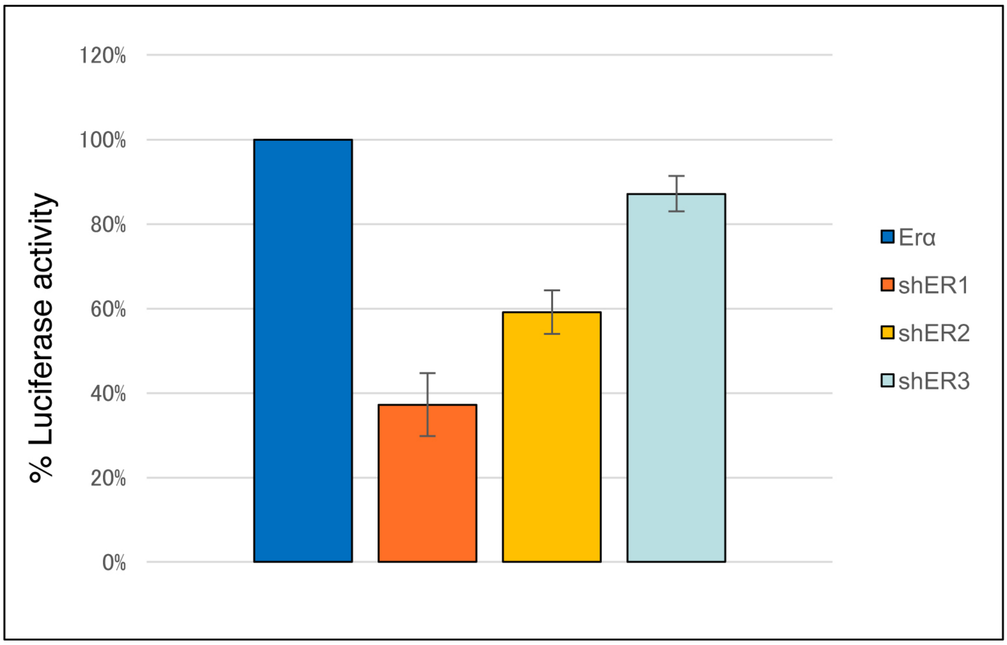

Development • Supplementary information

Fig. S6 Luciferase assay. (A) The diagram shows the activity of a Luciferase reporter under the control of a ERE enhancer in HEK293T cells co-transfected with *ERα* and the activator plasmid pT2K-CAG-rTA-M2 only (dark blue column), or in combination with the dominant negative form of *ERα* (*dnERα*) (orange column), or with the empty vector PT2K-B1-TRE-EGFP (light blue column) (see material and methods for plasmids reference). The luciferase bioluminescence was normalised to ß-galactosidase activity using ONPG. The activity of ERα alone (dark blue column) is taken as reference and set at 100%. Three biological replicates were performed in triplicate. The fold change is expressed as a percentage relative to the reference, with error bars showing the standard deviation (STD).

(B) The diagram shows the activity of a Luciferase reporter under the control of a ERE enhancer in HEK293T cells co-transfected with *ERα* in combination with psilencer2-U6 empty vector (dark blue column), or with *shER1* (orange column), or *shER2* (yellow column), or *shER3* (light blue column) cloned in psilencer. The luciferase bioluminescence was normalised to Renilla activity. The activity of ERα/psilencer2-U6 was taken as reference and set at 100% (dark blue column). The fold change is expressed as a percentage relative to the reference. Error bars show the STD of three biological replicates performed in triplicate.

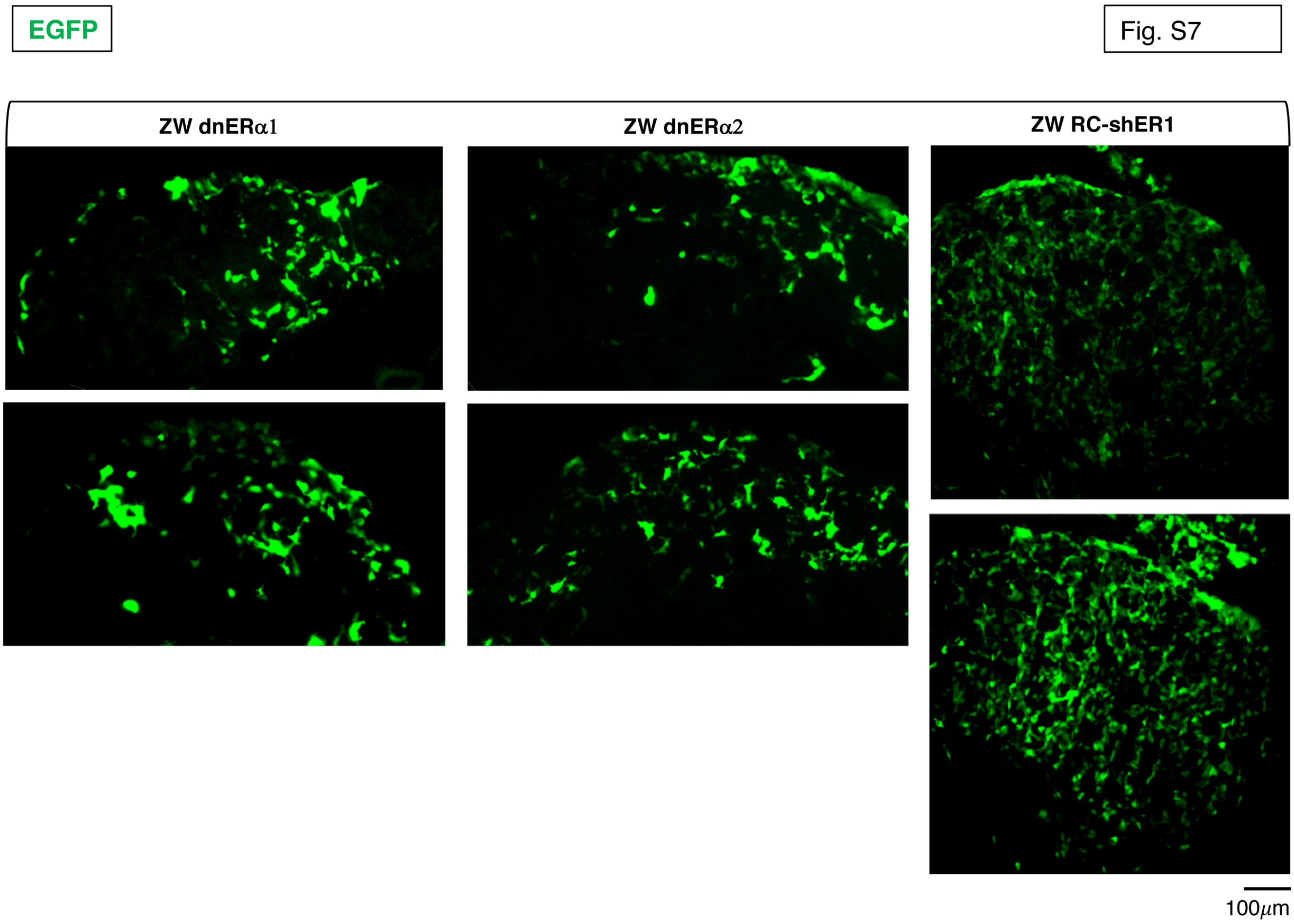

Fig. S7 EGFP reporter expression pattern in the sections from electroporated gonads shown in Fig. 6A-B.

Development • Supplementary information

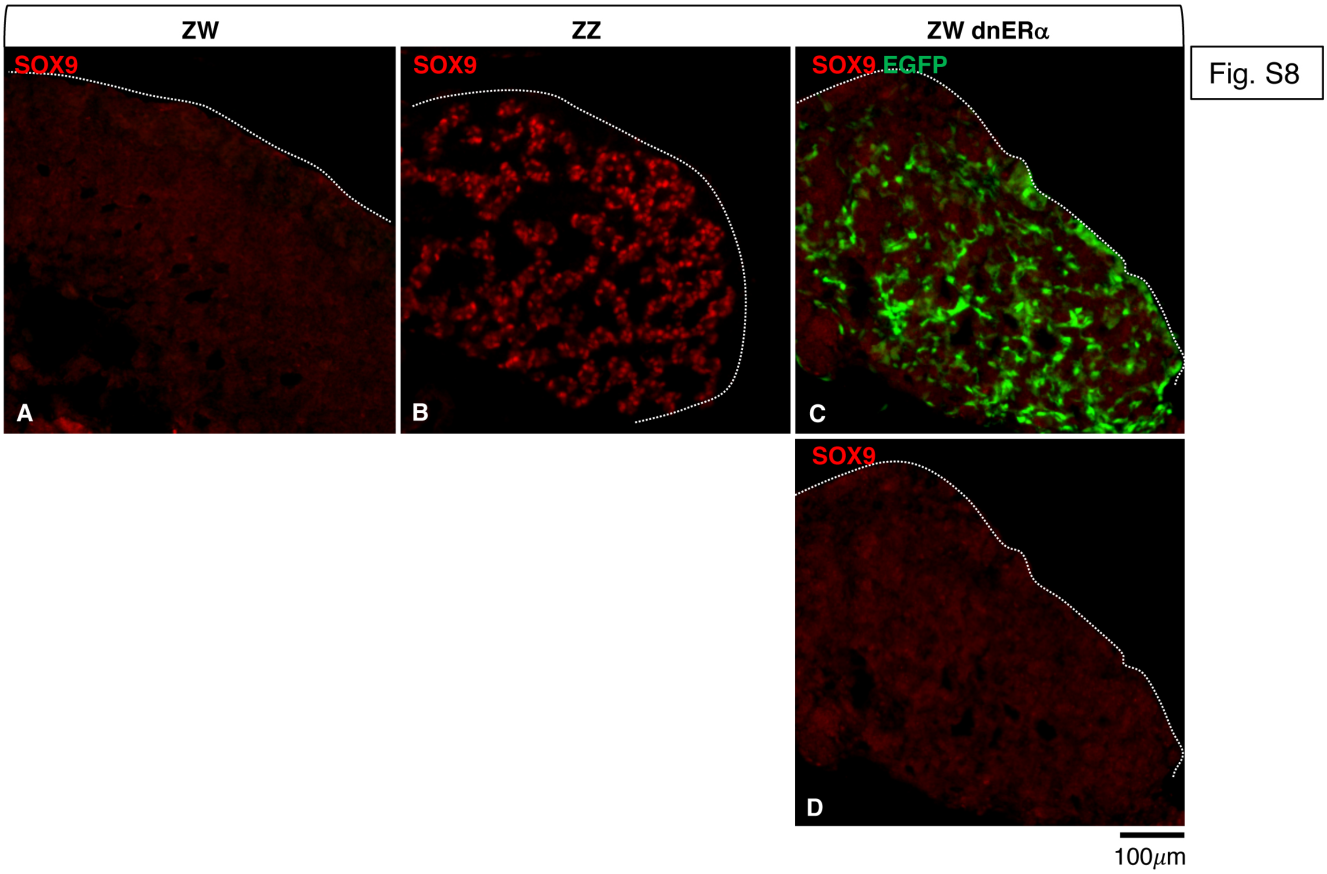

Fig. S8 The electroporation of dnERa into the left epithelium of ZW gonads does not change the medulla female identity. (A-D) Sections from D10 (HH36) gonads immunostained for the Sertoli marker SOX9 (red) in ZW control (A), ZZ control (B), ZW gonad electroporated with dnERα (C-D); dnERα targeted cells highlighted by EGFP reporter in (C). White dotted lines mark the edge of the gonad.

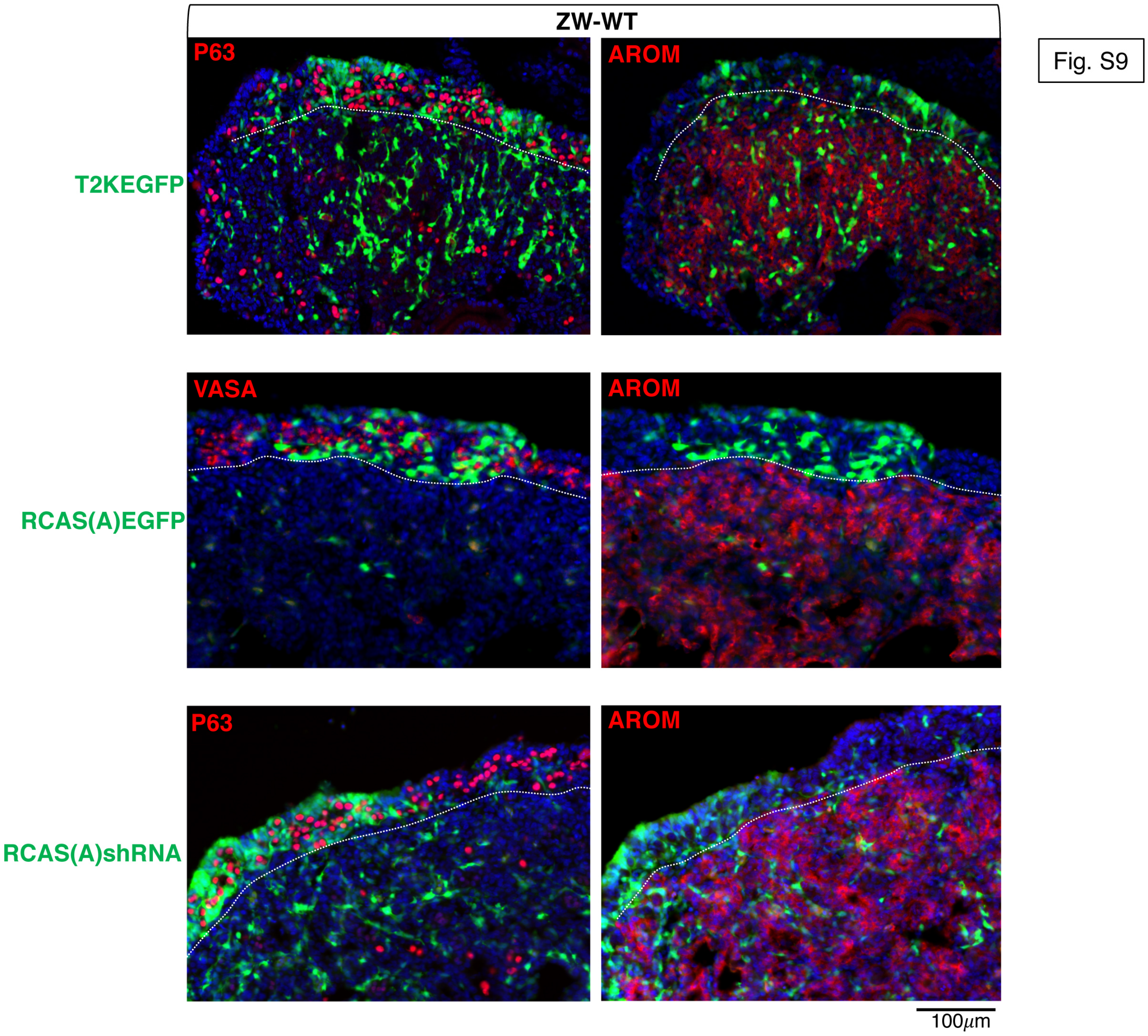

Fig. S9 Electroporation of the gonadal epithelium does not affect cortex formation. Immunofluorescent sections from D10 (HH36) ZW embryonic ovaries electroporated at D2.5 (HH15-17) with Tol2 control expression vectors T2K-EGFP (see Material and Methods), the retroviral vector RCAS(A) expressing EGFP [RCAS(A)EGFP], or RCAS(A) expressing EGFP and a shRNA targeting the gene *FOXL2* [RCAS(A)shRNA] (Oligo sequence: 5'-GGATCTACCAGTACATCAT-3'), which is not normally expressed in the epithelial cells. In red a germ cell marker (P63 or VASA) or P450aromatase (AROM); in green EGFP. White dotted lines show the cortex/medulla border. A minimum of 3 well electroporated embryos were analysed per construct.
